# Serum Autoantibodies against STIP1 as a Potential Biomarker in the Diagnosis of Esophageal Squamous Cell Carcinoma

**DOI:** 10.1155/2017/5384091

**Published:** 2017-08-09

**Authors:** Yi-Wei Xu, Can-Tong Liu, Xin-Yi Huang, Li-Sheng Huang, Yu-Hao Luo, Chao-Qun Hong, Hai-Peng Guo, Li-Yan Xu, Yu-Hui Peng, En-Min Li

**Affiliations:** ^1^Department of Clinical Laboratory Medicine, Cancer Hospital, Shantou University Medical College, Shantou 515041, China; ^2^Department of Biochemistry and Molecular Biology, Shantou University Medical College, Shantou 515041, China; ^3^The Key Laboratory of Molecular Biology for High Cancer Incidence Coastal Chaoshan Area of Guangdong Higher Education Institutes, Shantou University Medical College, Shantou 515041, China; ^4^Shantou University Medical College, Shantou 515041, China; ^5^Department of Radiation Oncology, Cancer Hospital, Shantou University Medical College, Shantou 515041, China; ^6^Department of Oncological Research Laboratory, Cancer Hospital, Shantou University Medical College, Shantou 515041, China; ^7^Department of Surgical Oncology, Cancer Hospital, Shantou University Medical College, Shantou 515041, China; ^8^Institute of Oncologic Pathology, Shantou University Medical College, Shantou 515041, China

## Abstract

Esophageal squamous cell carcinoma (ESCC) remains one of the leading causes of cancer-related mortality around the world. The identification of novel serum biomarkers is required for early detection of ESCC. This study was designed to elucidate whether autoantibodies against STIP1 could be a diagnostic biomarker in ESCC. An enzyme-linked immunosorbent assay was performed to detect serum levels of STIP1 autoantibodies in a training cohort (148 ESCC patients and 111 controls) and a validation cohort (60 ESCC patients and 40 controls). Mann–Whitney's *U* test showed that ESCC patients in two cohorts have higher levels of autoantibodies against STIP1 when compared to controls (*P* < 0.001). According to receiver operating characteristic analysis, the sensitivity, specificity, and area under the curve (AUC) of autoantibodies against STIP1 in ESCC were 41.9%, 90.1%, and 0.682 in the training cohort and 40.0%, 92.5%, and 0.710 in the validation cohort, respectively. Moreover, detection of autoantibodies against STIP1 could discriminate early-stage ESCC patients from controls, with sensitivity, specificity, and AUC of 35.7%, 90.1%, and 0.684 in the training cohort and 38.5%, 92.5%, and 0.756 in the validation cohort, respectively. Our findings indicated that autoantibodies against STIP1 might be a useful biomarker for early-stage ESCC detection.

## 1. Introduction

Esophageal cancer (EC) is the eighth most prevalent malignant disease and the sixth leading cancer-related deaths around the world [1]. In China, 477,900 EC patients were diagnosed and 375,000 patients died in 2015. Among them, the number of male patients was as twice as that of female patients [2]. Esophageal squamous cell carcinoma (ESCC) and esophageal adenocarcinoma are two predominant subtypes of EC. In China, 90% of cases are ESCC, compared to only 26% in the United States [3]. Despite many advances in the treatments of patients with EC, the 5-year survival rate remains poor (e.g., 17.4% in the United States) [4]. The survival rate of EC could reach up to 85% when diagnosed at an early stage but is no more than 10% if diagnosed at an advanced stage [5]. Thus, early diagnosis offers a great opportunity to receive effective therapy and reduce ESCC mortality, and the discovery of noninvasive screening methods is urgently needed.

Serological tests are found to be one of the promising methods for improvement of the early detection of cancer. However, ESCC lacks effective and reliable serological biomarker for early detection and disease surveillance. Actually, carcinoembryonic antigen (CEA), squamous cell carcinoma antigen (SCCA), and CYFRA21-1 were the most widely used serum biomarkers for ESCC, but the performance of these biomarkers to detect early-stage ESCC is deficient [6–8]. Novel biomarkers with high diagnostic accuracy are greatly needed to improve detection of ESCC. In the last decade, numerous studies have indicated that autoantibodies against tumor-associated antigens (TAAs), as reporters from the immune system, exist in cancer patients, and autoantibodies against TAAs are thought to be ideal targets as noninvasive serological tests for early detection of cancer [9–11]. Therefore, autoantibodies could be a valuable source of serum biomarkers used for identifying early ESCC.

Stress-induced phosphoprotein 1 (STIP1), also known as HOP, P60, STI1, and so forth, is a 66.2-kilodalton chaperone protein which plays important roles in stress and nonstress conditions. Its 2.0-kilobase-encoded mRNA was first isolated from the yeast *Saccharomyces cerevisiae* [12]. STIP1 is one of the cochaperones that are most extensively studied and contains three tetratricopeptide repeat (TPR) domains, which can simultaneously bind Hsp70 and Hsp90 [13, 14]. STIP1 was identified to be overexpressed in several kinds of cancers, such as colorectal carcinoma (CRC) [15], pancreatic cancer [16], cholangiocellular carcinoma (CCC) [17], ovarian cancer [18], and so on. Moreover, increased expression of STIP1 may indicate poor survival outcome in cancer patients [18, 19]. STIP1 was also identified as a TAA recognized by the humoral immune system by means of serological analysis of recombinant cDNA expression library approach [20]. A recent study showed that autoantibodies against STIP1 were significantly elevated in the serum levels of patients with ovarian cancer, compared with the normal controls [21]. However, no study has been conducted on STIP1 autoantibodies in esophageal cancer. The present study was then undertaken to investigate whether autoantibodies against STIP1 could be altered and used as a candidate diagnostic biomarker in ESCC.

## 2. Methods

### 2.1. Study Population

The serum samples of 148 patients with ESCC and 111 normal controls as a training cohort were collected from the Cancer Hospital of Shantou University Medical College, from March 2013 to June 2014. The sera of 40 normal controls and 60 ESCC patients obtained from the same hospital between July 2014 and February 2015 were used as a validation cohort. The sex and age were well matched in the patient group and control group in both cohorts (Table 1). ESCC patients were all newly diagnosed, and serum samples were obtained prior to any anticancer treatment. The normal controls must have no evidence of any malignancies based on physical examination. All patients and normal controls gave written informed consent to attend this study, which was approved by the institutional review board of the Cancer Hospital of Shantou University Medical College and conformed to the requirements of the Declaration of Helsinki.

ESCC was diagnosed and defined in our previous study [22, 23]. We defined tumor stage in accordance with the Seventh Edition of the *American Joint Committee on Cancer (AJCC) Cancer Staging Manual* [24]. In the present work, ESCC with AJCC stage 0 + I + IIA was treated as early-stage ESCC as reported [22].

### 2.2. Recombinant STIP1 Protein

The expression, purification, and analysis of the recombinant STIP1 proteins were conducted as previously described [22, 23]. Briefly, the coding sequence region of STIP1 (NM_006819) was subcloned into the pDEST17 vector system (Invitrogen). The recombinant plasmid was transformed into the expression host *E. coli* Rosetta (DE3; Novagen). Transformed colonies were inoculated and cultured overnight. Then, the cell culture was transferred to fresh LB medium. To induce expression of recombinant protein, IPTG (Merck) was added. Next, the cells were harvested and resuspended. Cell debris was cleared by centrifugation, and the supernatants were incubated by using a Ni2þ-NTA-Sepharose column (Novagen). The proteins of interest were eluted and dialyzed. BCA protein assay (Thermo) was performed to determine protein concentrations with the use of bovine serum albumin as a standard. The purity of the recombinant protein was assessed by Coomassie Blue staining (Imperial Protein Stain; Thermo) following SDS-PAGE.

### 2.3. Enzyme-Linked Immunosorbent Assay (ELISA)

ELISA was used to detect serum levels of autoantibodies against STIP1 by two investigators (Can-Tong Liu and Xin-Yi Huang) who were blinded to clinical information about ESCC patients and normal controls. ELISA protocol was conducted according to our prior work [22, 25]. In brief, recombinant STIP1 protein diluted to a concentration of 0.2 *μ*g/mL was dispensed in 100 *μ*L per well volumes into 96-well microtiter plates and incubated overnight at 4°C. The protein-coated wells were blocked with PBST containing 0.05% Tween-20 and 1% BSA at 37°C for 1 h. After washing, serum samples and self-made quality control samples (a pooled serum sample randomly collected from 100 ESCC patients), all at the dilution of 1 : 110, were incubated at 37°C for another one hour, as well as appropriate polyclonal rabbit anti-STIP1 antibodies (Immunosoft, Zhoushan, China) specific for captured proteins. 100 *μ*L of secondary antibodies (i.e., goat anti-human/anti-mouse IgG-HRP) diluted at 1 : 10,000 was added into each well, followed by color development (ready-prepared 3,3′,5,5′-tetramethylbenzidine and hydrogen peroxide). The measurement of optical density (OD) value of each well was completed on a microplate reader (Multiskan MK3, Thermo Fisher Scientific, Boston) within 5 min at 450 nm with 630 nm reference.

All serum samples were tested in duplicate. Quality control for monitoring of intra-assay deviation or interassay deviation of the ELISA assay was conducted as described in our previous studies [22, 25]. Briefly, the intra-assay and interassay coefficient of variations (CVs) for the ELISA method of the detection of autoantibodies against STIP1 were 7.9% and 9.4%, respectively. Quality control samples were conducted to ensure quality control monitoring of the assay runs with the use of Levey-Jennings plots. To minimize an intra-assay deviation, the ratio of the difference between the duplicated sample OD values to their sum was used to evaluate assay precision. If the ratio was >10%, the measurement of this sample was deemed to be invalid and the sample was repeated.

### 2.4. Statistical Analysis

We performed all analyses by using Microsoft Excel, GraphPad Prism 5 software, and SPSS (version 17.0). The results of sample positive proportion and diagnostic parameters were shown with 95% exact confidence interval (95% CI) estimation. The differences of serum autoantibody levels between patients and controls were compared using the Mann–Whitney *U* test; the positive rates of STIP1 autoantibodies between the patient group and control group or in each group of patients' sera were compared using chi-squared tests. We plotted receiver operating characteristic (ROC) curve to discuss the sensitivity, specificity, and area under the ROC curve (AUC). The cutoff value for autoantibody positivity was determined by achieving the maximum sensitivity when the specificity was more than 90% and by minimizing the distance of the cutoff value to the top-left corner of the ROC curve (all ESCC versus all normal controls in the training cohort). The test result indicated a statistically significant difference when *P* value was less than 0.05 (two-sided).

## 3. Results

### 3.1. Level of Serum Autoantibodies against STIP1

**Figure 1:**
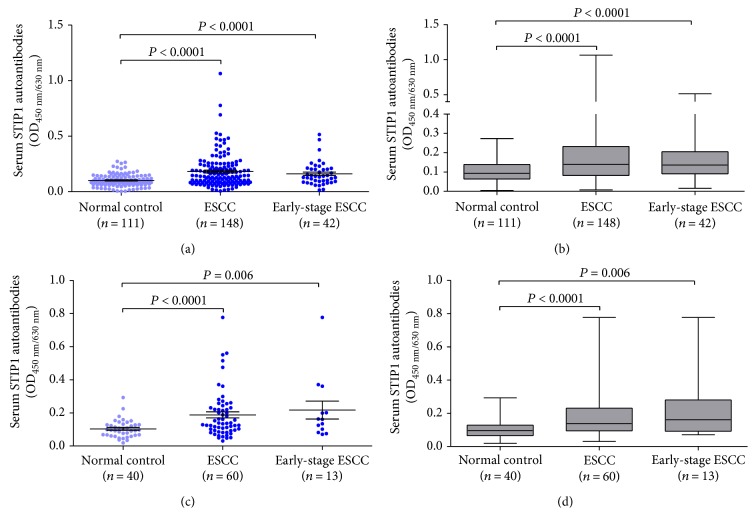
Levels of autoantibodies against STIP1 in ESCC. (a) Scatter plots of OD values of autoantibodies against STIP1 from sera of normal controls, ESCC patients, and early-stage ESCC patients in the training cohort. Black horizontal lines are means and error bars are SEs. (b) Median levels and interquartile ranges of serum autoantibodies against STIP1 in normal controls, ESCC patients, and early-stage ESCC patients in the training cohort are, respectively, illustrated by box plot, and the whiskers show minimum and maximum value. (c) Scatter plots of OD values of autoantibodies against STIP1 from sera of normal controls, ESCC patients, and early-stage ESCC patients in the validation cohort. Black horizontal lines are means and error bars are SEs. (d) Median levels and interquartile ranges of serum autoantibodies against STIP1 in normal controls, ESCC patients, and early-stage ESCC patients in the validation cohort are, respectively, illustrated by box plot, and the whiskers show minimum and maximum value.

In the group of all ESCC patients in the training cohort, the mean OD ± SD of STIP1 autoantibodies was 0.182 ± 0.150 and was 0.161 ± 0.105 and 0.102 ± 0.056 in the early-stage patient group and normal control group, respectively. We observed that patients with ESCC had a significant increase in level of serum STIP1 autoantibody, compared with normal controls (Figure 1, *P* < 0.0001). As shown in Figure 1, similar result was noted in early-stage ESCC patients (*P* < 0.0001). In the validation cohort, serum STIP1 autoantibody levels were also raised in ESCC patients, compared with controls (Figure 1).

### 3.2. Diagnostic Performance of Serum Autoantibodies against STIP1

**Figure 2:**
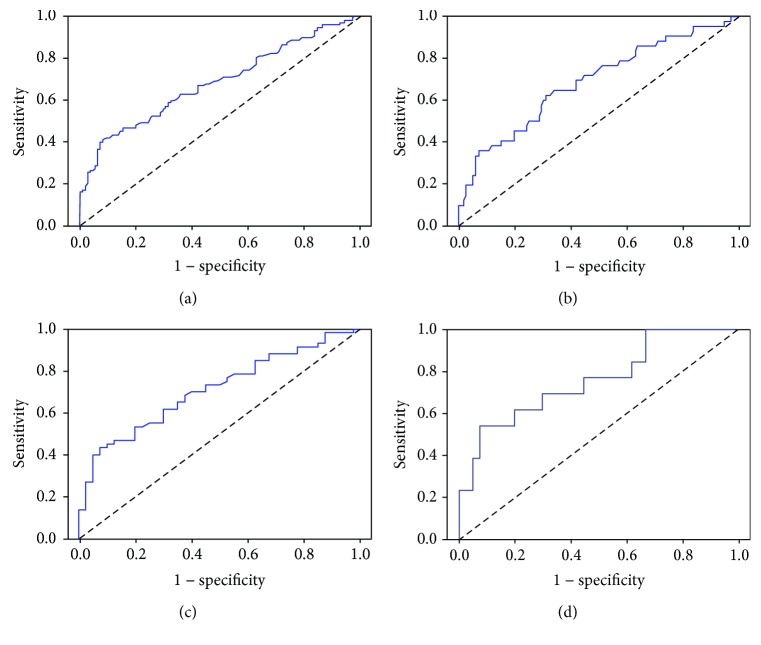
Receiver operating characteristic (ROC) curve analysis in the diagnosis of esophageal squamous cell carcinoma (ESCC). (a) ROC curve for serum autoantibodies against STIP1 for patients with ESCC versus normal controls in the training cohort. (b) ROC curve for serum autoantibodies against STIP1 for patients with early-stage ESCC versus normal controls in the training cohort. (c) ROC curve for serum autoantibodies against STIP1 for patients with ESCC versus normal controls in the validation cohort. (d) ROC curve for serum autoantibodies against STIP1 for patients with early-stage ESCC versus normal controls in the validation cohort.

Using ROC analysis (all ESCC group versus control group in the training cohort), we identified a cutoff value of 0.173 for serum autoantibodies against STIP1 to diagnose ESCC (Figure 2). In both cohorts, the positive rates of serum autoantibodies against STIP1 increased not only in patients with ESCC but also in the early-stage patients, compared with normal controls (*P* < 0.0001, Table 2). For all ESCC patients in the training cohort, autoantibodies against STIP1 had an AUC of 0.682 (95% CI: 0.619–0.746) to distinguish individuals with ESCC from normal controls with a sensitivity/specificity of 41.9% (95% CI: 33.9%–50.3%)/90.1% (95% CI: 82.6%–94.7%) (Figure 2, Table 3). Autoantibodies against STIP1 also identified early-stage ESCC with a similar AUC value of 0.684 (95% CI: 0.586–0.782), a sensitivity of 35.7% (95% CI: 22.0–52.0%), and a specificity of 90.1% (95% CI: 82.6%–94.7%). In the validation cohort, we found similar diagnostic performance to those from the training cohort when the same cutoff value was used (Table 3). To improve clinical interpretation, we also presented false positive rate, false negative rate, predictive values, and likelihood ratios for autoantibodies against STIP1 in ESCC diagnosis in Table 3.

### 3.3. Correlation between Autoantibodies against STIP1 and ESCC Clinicopathological Variables

Tables 4 and 5 demonstrate the relationship of the levels of autoantibodies against STIP1 with clinicopathological features in ESCC in the training cohort and validation cohort, respectively. However, serum levels of autoantibodies against STIP1 showed no significant associations with clinicopathologic variables examined, including patient age, patient gender, size of tumor, site of tumor, histological grade, lymph node status, or early-stage and advanced-stage groups (all *P* > 0.05).

## 4. Discussion

Until now, it has been destitute of efficient early detection approach for ESCC, leading to a postponement of diagnosis and treatment in the majority of patients. To improve long-term survival and life quality of cancer patients, early detection remains the most promising approach. Endoscopic screening has been proven to be a valid method to detect early ESCC and can decrease incidence and mortality of ESCC [26]. But it still could not be widely used as a screening tool for esophageal cancer due to its invasive nature. Therefore, we need to identify serum/plasma biomarkers that could effectively detect early ESCC. In the present study, the serum levels of autoantibodies against STIP1 were significantly increased in ESCC patients compared with normal controls. In addition, autoantibodies against STIP1 could detect early-stage ESCC (AJCC 0 + I + IIA). Our findings indicated autoantibodies against STIP1 as a potential noninvasive biomarker for early ESCC detection.

In recent years, there is no doubt that autoantibodies against TAAs have been a hot topic of research in early cancer diagnosis. Since autoantibodies against TAA in the sera of melanoma patients were first reported [27], a huge number of autoantibodies have been reported as early diagnostic biomarkers for cancers [9–11, 21–23, 28–30]. EarlyCDT-Lung, as a convenient blood test measuring autoantibodies against seven TAAs, shows the ability to aid in risk assessment and the early detection of lung cancer in high-risk, asymptomatic patients [31, 32]. In ESCC, there are also findings related to measurement of a panel of autoantibodies [22, 33]. Our prior work using a panel of six TAAs (i.e., p53, NY-ESO-1, MMP-7, Hsp70, PRDX, and Bmi-1) to assess the early-stage ESCC detection obtained a sensitivity and specificity of 45% and 95%, respectively [22]. Zhang et al. also reported that autoantibodies against a panel of four TAAs (i.e., c-Myc, HCCR, p53, and p62) provided a high diagnostic efficiency for early-stage ESCC detection [33]. All the abovementioned studies highlight the significance of the combined measurement of an optimized autoantibody panel in the diagnosis of early cancer. However, the two panels of TAAs that have been reported do not have high enough sensitivity as a reliable screening test for early ESCC [22, 33]. This study shows that autoantibodies against STIP1 have sensitivities of 35.7% (95% CI: 22.0–52.0%) in the training cohort and 38.5% (95% CI: 15.1%–67.7%) in the validation cohort to diagnose early-stage ESCC. Such sensitivity is better than the recently identified autoantibody biomarkers in early-stage ESCC reported by our team, including autoantibodies against ezrin, fascin, and L1CAM [23, 34, 35], which indicates that autoantibodies against STIP1 might be an encouraging candidate for establishment of an optimized autoantibody signature required to gain high sensitivity necessary for ESCC screening. In the next stage of the research, we will further evaluate whether the combination of autoantibodies against STIP1 with other autoantibody targets would increase the diagnostic sensitivity of our previously reported autoantibody panel [22].

To date, there is not yet relevant research of STIP1 in esophageal cancer. This study provides evidence that STIP1 induced autoantibody responses in sera of ESCC patients. It is generally believed that autoantibody production is involved with alterations in expression level, degradation, or posttranslational modification of cellular proteins [36]. Though what mechanism might underlie the production of autoantibodies against STIP1 in esophageal cancer is unclear, our findings provide important clues for further study of biological function of STIP1 in ESCC. In future work, we would perform a systematic study, including in vitro cell-based research and animal experiments, to reveal the function and molecular mechanisms of STIP1 in esophageal cancer. We could suppose that STIP1 might play a role in the carcinogenesis and development of ESCC.

## 5. Conclusion

To our knowledge, this is the first study that shows the elevated levels of serum autoantibodies against STIP1 in ESCC patients. The diagnostic value of serum autoantibodies against STIP1 for ESCC was verified in the training cohort and in an independent validation cohort. Our data demonstrated that autoantibodies against STIP1 might be a potential biomarker significantly associated with ESCC. However, the sample size of patients with early-stage ESCC was relatively small in this study. Further validation of the diagnostic value of autoantibodies against STIP1 in larger sample set is warranted.

## Figures and Tables

**Table 1:** Participant details and clinicopathological features.

Group	Training cohort		Validation cohort	
	ESCC (*n* = 148)	Normal (*n* = 111)	ESCC (*n* = 60)	Normal (*n* = 40)
Age, years				
Mean ± SD	58 ± 9	57 ± 8	59 ± 6	56 ± 7
Range	41–88	38–77	43–75	40–71
Gender				
Male	114	69	38	26
Female	34	25	22	14
Smoke				
Yes	106	73	36	29
No	42	38	24	11
TNM stage				
0	3		1	
I (IA + IB)	17 (7 + 10)		8 (3 + 5)	
II (IIA + IIB)	48 (22+ 26)		20 (4 + 16)	
III (IIIA + IIIB + IIIC)	76 (32+ 14+ 30)		30 (15+ 2 + 13)	
IV	4		1	
Histological grade				
High (grade 1)	50		17	
Middle (grade 2)	84		36	
Low (grade 3)	14		7	
Depth of tumor invasion				
Tis	3		1	
T1	11		10	
T2	19		11	
T3	77		20	
T4	38		18	
Regional lymph nodes				
N0	77		31	
N1	38		19	
N2	21		9	
N3	12		1	
Size of tumor				
<5 cm	71		24	
≥5 cm	77		36	
Site of tumor				
Upper thorax	15		10	
Middle thorax	101		46	
Lower thorax	32		4	

**Table 2:** Frequency of autoantibodies against STIP1.

Group	*N*	Positive (%, 95% CI)	*P* value
Training cohort			
All ESCC	148	62 (41.9%, 33.9%–50.3%)	<0.0001
Early-stage ESCC (0 + I + IIA)	42	15 (35.7%, 22.0%–52.0%)	<0.0001
Normal controls	111	11 (9.9%, 5.3%–17.4%)	
Validation cohort			
All ESCC	60	24 (40.0%, 27.8%–53.5%)	<0.0001
Early-stage ESCC (0 + I + IIA)	13	5 (38.5%, 15.1%–67.7%)	<0.05
Normal controls	40	3 (7.5%, 2.0%–21.5%)	

ESCC: esophageal squamous cell carcinoma; *P* value is relative to normal controls. Statistical significance was determined using unpaired chi-square test.

**Table 3:** Results for measurement of the STIP1 autoantibodies in the diagnosis of ESCC.

	AUC	SEN	SPE	FPS	FNS	PPV	NPV	PLR	NLR
Training cohort									
ESCC versus NC	0.682 (0.619–0.746)	41.9% (33.9%–50.3%)	90.1% (82.6%–94.7%)	9.9% (5.3%–17.4%)	58.1% (49.7%–66.1%)	84.9% (74.2%–91.9%)	53.8% (46.3%–61.0%)	4.23 (2.34–7.64)	0.64 (0.56–0.74)
Early-stage ESCC versus NC	0.684 (0.586–0.782)	35.7% (22.0%–52.0%)	90.1% (82.6%–94.7%)	9.9% (5.3%–17.4%)	64.3% (48.0%–78.0%)	57.7% (37.2%–76.0%)	78.7% (70.4%–85.3%)	3.60 (1.80%-7.20)	0.71 (0.57%-0.90)
Validation cohort									
ESCC versus NC	0.710 (0.610–0.810)	40.0% (27.8%–53.5%)	92.5% (78.5%–98.0%)	7.5% (2.0%–21.5%)	60.0% (46.5%–72.2%)	88.9% (69.7%–97.1%)	50.7% (38.8%–62.5%)	5.33 (1.72–16.54)	0.65 (0.53–0.80)
Early-stage ESCC versus NC	0.756 (0.598–0.913)	38.5% (15.1%–67.7%)	92.5% (78.5%–98.0%)	7.5% (2.0%–21.5%)	61.5% (32.3%–84.9%)	62.5% (25.9%–89.8%)	82.2% (67.4%–91.5%)	5.13 (1.42–18.58)	0.67 (0.43–1.03)

All values are given with 95% CI in each group. ESCC: esophageal squamous cell carcinoma; NC: normal controls; AUC: area under the ROC curve; SEN: sensitivity; SPE: specificity; FPS: false positive rate; FNS: false negative rate; PPV: positive predictive value; NPV: negative predictive value; PLR: positive likelihood ratio; NLR: negative likelihood ratio.

**Table 4:** Relationship between positive rate of the STIP1 autoantibodies and clinical data in ESCC patients from the training cohort.

	*N*	Positive (%, 95% CI)	*P*
Age			
≥58	84	40 (47.6, 36.7–58.7)	0.106
<58	64	22 (34.4, 23.3–47.4)	
Gender			
Male	114	49 (43.0, 33.8–52.6)	0.622
Female	34	13 (38.2, 22.7–56.4)	
Smoke			
Yes	106	45 (42.5, 33.0–52.4)	0.826
No	42	17 (40.5, 26.0–56.7)	
Site of tumor			
Upper thorax	15	7 (46.7, 22.3–72.6)	0.880
Middle thorax	101	41 (40.6, 31.1–50.8)	
Low thorax	32	14 (43.8, 26.8–62.1)	
Size of tumor			
<5 cm	71	32 (45.1, 33.4–57.3)	0.452
≥5 cm	77	30 (39.0, 28.3–50.8)	
Depth of tumor invasion			
T1 + T2	30	9 (30.0, 15.4–49.6)	0.133
T3 + T4	115	52 (45.2, 36.0–54.8)	
Regional lymph nodes			
N0	77	30 (39.0, 28.3–50.8)	0.452
N1 + N2 + N3	71	32 (45.1, 33.4–57.3)	
Histological grade			
G1	50	18 (36.0, 23.3–50.9)	0.440
G2	84	39 (46.4, 35.6–57.6)	
G3	14	5 (35.7, 14.0–64.4)	
TNM stage			
Early stage (0 + I + IIA)	42	15 (35.7, 22.0–52.0)	0.338
Advanced stage (IIB + III + IV)	106	47 (44.3, 34.8–54.3)	

ESCC: esophageal squamous cell carcinoma; CI: exact confidence interval. Statistical significance was determined using the chi-square test.

**Table 5:** Relationship between positive rate of the STIP1 autoantibodies and clinical data in ESCC patients from the validation cohort.

	*N*	Positive (%, 95% CI)	*P*
Age			
≥58	32	13 (40.6, 24.2–59.2)	0.916
<58	28	11 (39.3, 22.2–59.3)	
Gender			
Male	38	14 (36.8, 22.3–54.0)	0.512
Female	22	10 (45.5, 25.1–67.3)	
Smoke			
Yes	36	13 (36.1, 21.3–53.8)	0.451
No	24	11 (45.8, 26.2–66.8)	
Site of tumor			
Upper thorax	10	3 (30.0, 8.1–64.6)	0.724
Middle thorax + low thorax	50	21 (42.0, 28.5–56.7)	
Size of tumor			
<5 cm	24	9 (37.5, 19.6–59.2)	0.747
≥5 cm	36	15 (41.7, 26.0–59.1)	
Depth of tumor invasion			
T1 + T2	21	9 (42.9, 22.6–65.6)	0.800
T3 + T4	38	15 (39.5, 24.5–56.5)	
Regional lymph nodes			
N0	31	12 (38.7, 22.4–57.7)	0.833
N1 + N2 + N3	29	12 (41.4, 24.1–60.9)	
Histological grade			
G1	17	6 (35.3, 15.3–61.4)	0.659
G2	36	16 (44.4, 28.3–61.7)	
G3	7	2 (28.6, 5.1–69.7)	
TNM stage			
Early stage (0 + I + IIA)	13	5 (38.5, 15.1–67.7)	0.898
Advanced stage (IIB + III + IV)	47	19 (40.4, 26.7–55.7)	

ESCC: esophageal squamous cell carcinoma; CI: exact confidence interval. Statistical significance was determined using the chi-square test.
